# Omega-3 Polyunsaturated Fatty Acids and Cognitive Decline in Adults with Non-Dementia or Mild Cognitive Impairment: An Overview of Systematic Reviews

**DOI:** 10.3390/nu17183002

**Published:** 2025-09-19

**Authors:** Maria Inês Barros, Teresa Brandão, Susana Couto Irving, Paula Alves, Filomena Gomes, Marta Correia

**Affiliations:** 1 Hospital Santa Maria—Porto, 4049-025 Porto, Portugal; mariaines.barros@hsmporto.pt; 2 CBQF—Centro de Biotecnologia e Química Fina, Universidade Católica Portuguesa, 4169-005 Lisboa, Portugal; tbrandao@ucp.pt; 3 Instituto Português Oncologia do Porto Francisco Gentil, EPE, 4200-162 Porto, Portugal; 4 NOVA Medical School, Universidade Nova de Lisboa, 1169-056 Lisboa, Portugal

**Keywords:** Alzheimer’s disease, dementia, mild cognitive impairment/decline, omega-3 polyunsaturated fatty acids supplementation

## Abstract

Background/Objectives: As global aging accelerates, prevalence of mild cognitive impairment (MCI) continues to rise, challenging healthcare systems and diminishing older adults’ quality of life. There is great interest in better understanding the neuroprotective/anti-inflammatory properties of omega-3 polyunsaturated fatty acids but the results from many published studies in humans come to different conclusions. This review aims to clarify the efficacy of *n*-3 fatty acids as a preventive or therapeutic strategy for cognitive health and to inform future clinical recommendations within aging populations. Methods: Following PRISMA guidelines and a registered PROSPERO protocol, we reviewed systematic reviews (SRs) from 2014 to 2024 assessing exclusive *n*-3 fatty acid supplementation and cognitive outcomes via MMSE. Data were extracted on intervention details and cognitive scores. Meta-analyses used fixed and random-effects models, with Hedges’ estimating overall impact. Quality was assessed using AMSTAR-2, and statistical analyses were performed (SPSS 28). Results: A total of nine SRs incorporating 14 RCTs were included, representing 26,881 participants aged 40 years or older. The pooled random-effects meta-analysis showed a statistically significant but modest improvement in MMSE scores (effect size: 0.16; 95% CI: 0.01–0.32). Heterogeneity was moderate (I^2^ = 42.8%), and no publication bias was detected. Further analyses revealed no significant associations between treatment duration or dosage and cognitive outcomes, suggesting a threshold effect rather than a dose–response relationship. Conclusions: These findings support n3-PUFA supplementation as a complementary approach to lifestyle-based strategies for cognitive health, including diet, physical activity, sleep optimization, and cognitive training. While benefits appear modest, consistent effects across studies warrant further high-quality research and well-designed studies to strengthen clinical recommendations.

## 1. Introduction

As the global population ages, the prevalence of cognitive decline, including MCI, continues to rise, challenging worldwide healthcare systems [1,2]. MCI is an initial stage of memory loss or decline in other cognitive functions in individuals who can still perform most daily activities independently and affects 12 to 18% of people aged 60 or older, representing a major cause of the decrease in quality of life among older adults [3,4]. The Mini Mental State Examination (MMSE) is a tool widely used to assess cognitive function and is strongly associated with dementia diagnosis, as lower MMSE scores indicate a higher likelihood of developing dementia [5,6,7].

Lifestyle modifications including balanced nutrition, physical activity, and cognitive stimulation are associated with reduced risk of cognitive decline through improved neuroplasticity, vascular health, and reduced oxidative stress [8,9]. N3 PUFA, particularly EPA and DHA, play essential roles in synaptic integrity, membrane fluidity, and anti-inflammatory signaling, potentially enhancing cognitive function [10,11,12,13,14]. The neuroprotective effects include mechanisms that reduce neuroinflammation, enhance synaptic function, and support neuronal membrane integrity [15]. However, as previously pointed out, outcomes on whether supplementation with n3-PUFAs improve cognitive outcomes amongst non-demented individuals or those with MCI have been found across studies, likely due to differences in dosage, supplementation duration, baseline nutritional status, and genetic factors [14,16,17]. No meta-analysis has conclusively demonstrated that its supplementation leads to a significant cognitive improvement (irrespective of dosage or duration) [14,18,19]. Thus, while *n*-3 PUFAs show promising neuroprotective agents, optimizing their efficacy requires personalized approaches integrating lifestyle, clinical, and biomarker-based assessments. Importantly, variability in the study designs, participant demographics, and the intervention protocols might explain part of the inconsistencies found across the literature [18,19,20].

The aim of this umbrella review is to provide a comprehensive evaluation of existing SRs and meta-analyses on the effect of *n*-3 PUFA intervention on cognition in healthy individuals and individuals with mild cognitive decline or impairment. By systematically analyzing findings across different studies, this review aims to clarify whether n3-PUFA supplementation can be recommended as a viable approach for cognitive health maintenance or to prevent further cognitive decline. The findings will help guide future research directions and inform clinical recommendations, particularly as aging populations continue to grow.

## 2. Materials and Methods

### 2.1. Study Design

The study protocol for this review was registered on PROSPERO, registration number CRD42024552418. The Preferred Reporting Items for Systematic Reviews and Meta-Analyses (PRISMA) statement was used to identify, screen, evaluate, and include papers for this review [21,22].

### 2.2. Data Sources and Search Strategy

The literature searches were conducted in MEDLINE, Cochrane Database of Systematic Reviews, Scopus, and PROSPERO International prospective register of SRs between January 2014 and December 2024. The MeSH terms were (“fish oil” OR “EPA” OR “DHA” OR “omega 3 fatty acids) AND (“dementia” OR “Alzheimer’s disease” OR “mild cognitive impairment” OR “mild cognitive decline” OR “cognitive impairment” OR “cognitive decline”) with the filters (systematic reviews) OR (meta-analysis). Selected publication languages were English, Spanish, and Portuguese. We included SRs of RCT randomized clinical controlled trials (RCTs) to assess the putative effects of the abovementioned intervention on cognition, memory, attention, and concentration using specific tests for diagnosing cognitive impairment stages recommended by major diagnostic guidelines and scientific societies. No observational studies (including cohort and case–control studies) were included. This umbrella review has excluded RCTs that have used any other nutrient, or a mixture of other nutrients, as well as nutraceuticals or any other molecule besides n3-PUFAs. The titles and abstracts of all identified reviews were screened by two independent assessors (MIB, MMC) against the inclusion and exclusion criteria. Disagreements between the reviewers were resolved through discussion with a third reviewer (SCI).

### 2.3. Eligibility Criteria

**Figure 1:**
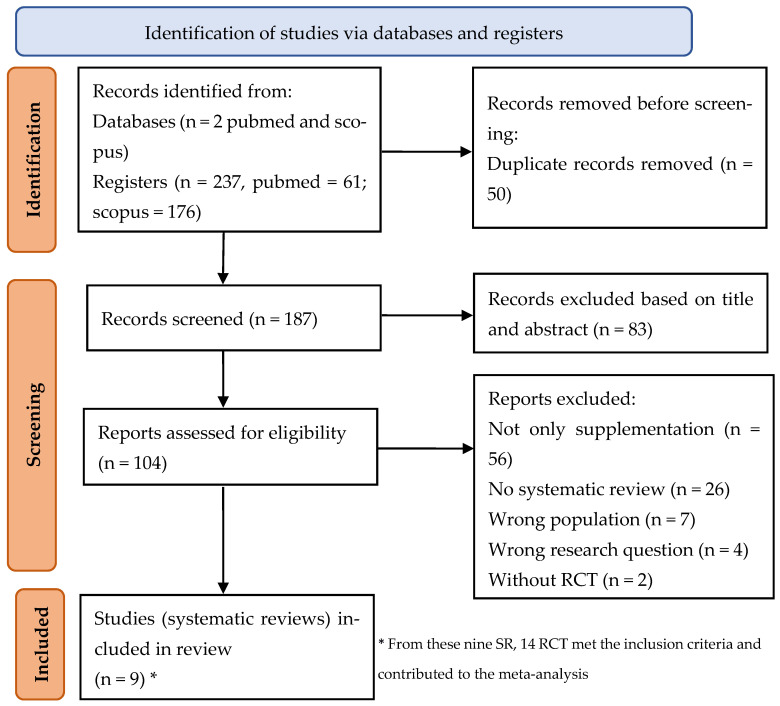
Flow diagram of the search strategy according to PRISMA 2020 [21,22].

The selection/exclusion criteria are in Table 1, and the process of included studies is summarized in a PRISMA 2020 flow diagram (Figure 1) [21,22].

After identifying eligible SRs, we proceeded with the data extraction, including authors, year of publication, objective of the review, number of RCT included, number of patients, interventions, comparators, time of intervention, outcomes, results, and conclusions. We considered the level of cognitive impairment as assessed by the MMSE as the primary outcome [17].

The MMSE cut-off values are as follows: 24 and higher: normal cognition; 19–23: MCI; 10–18: moderate cognitive impairment; and 9 and lower: severe cognitive impairment. A score of 23 or below on the MMSE’s 30-point scale is considered indicative of dementia [6]. While we acknowledge the influence of educational level on cognitive assessment scores, this factor was not explicitly mentioned as a criterion in the articles reviewed for our study. Consequently, we did not incorporate it in our inclusion/exclusion criteria.

### 2.4. Data Extraction

Data extracted from included meta-analyses encompass authors, year, title, population, characteristics, duration of the intervention, the supplementation dose, and MMSE results, 95% CI. For each SR we selected only the trials that met our inclusion criteria. It was important to select the trials that assessed cognitive impairment with the validated tool MMSE, allowing us to conduct meta-analyses of eligible trials. Thus, we analyzed the effect of n3-PUFA supplementation across multiple studies, using random-effects and fixed-effects models to estimate the overall effect of EPA and DHA supplementation on cognitive impairment.

### 2.5. Quality Assessment

The methodological quality of all the reviews included was assessed by two independent assessors (MIB, MMC) using AMSTAR-2 [23]. This tool involves sixteen items, seven critical, and rates the quality as high, moderate, low, or critically low, according to the number of critical flaws or non-critical weaknesses. We had three reviews classified as high, three as moderate, one as low, and two as critically low.

### 2.6. Statistical Analysis

The meta-analysis was conducted using both random-effects and fixed-effects models to estimate the overall effect of EPA and DHA supplementation on cognitive impairment. Hedges’ g and its 95% confidence intervals were calculated to provide a pooled estimate of the standardized mean difference across all included studies.

Between-study heterogeneity was assessed using both the I^2^ statistic and Cochran’s Q test. I^2^ values of 25%, 50%, and 75% were interpreted as indicative of low, moderate, and high heterogeneity, respectively. Publication bias and small-study effects were evaluated using Egger’s test and visual inspection of funnel plots.

To explore possible sources of variability in effect size, Pearson correlation analyses were conducted to examine associations between Hedges’ g and both treatment duration and EPA + DHA dosage.

All data analyses were performed using IBM SPSS Statistics, version 27.0 (IBM Corp., Armonk, NY, USA), with a significance level set at 5%.

## 3. Results

Out of 237 eligible articles, 104 articles were read in full (PRISMA Figure 1). Finally, nine SRs were included in the review reporting (Table 2). From these, 14 trials met the inclusion criteria and contributed to the meta-analyses.

The publication years ranged from 2014 until 2025, with four European and five Chinese articles.

Table 2 and Table 3 summarize the characteristics of the included SRs. A total of 26,881 individuals, 40 years or older, participated in the clinical trials. Various tools were used to analyze cognitive function (e.g., MMSE, Wechsler Adult Intelligence Scale, Montreal Cognitive Assessment scale (MoCA)), with MMSE being the most used tool. To remove the heterogeneity associated with the use of multiple tools or tests for assessment of cognitive function, we limited the inclusion criteria of RCTs to those that used MMSE, enabling us to perform meta-analysis and quantify the size effect of the intervention.

### 3.1. Effects of n3PUFA Supplementation on Cognitive and Functional Status

The effects of supplementation on cognitive function were consistent in most SRs. Seven SRs reported an improvement in the MMSE scores in the intervention group compared to the control, and only one reported no effect with *n*-3 PUFA supplementation. The following tables (Table 4 and Table 5) describe the 14 included RCTs that contributed to the meta-analysis [31,32,33,34,35,36,37,38,39,40,41,42,43]. RCTs were conducted in different countries and settings which enhances the generalizability of the findings.

### 3.2. Pooled Data on Cognitive Impairment (Assessed with MMSE from RCTs)

After comparing the results of the meta-analyses using random-effects and fixed-effects models to estimate the overall effect of EPA and DHA supplementation on cognitive impairment and realizing that similar results were obtained, we decided to display only the random effects models.

**Figure 2:**
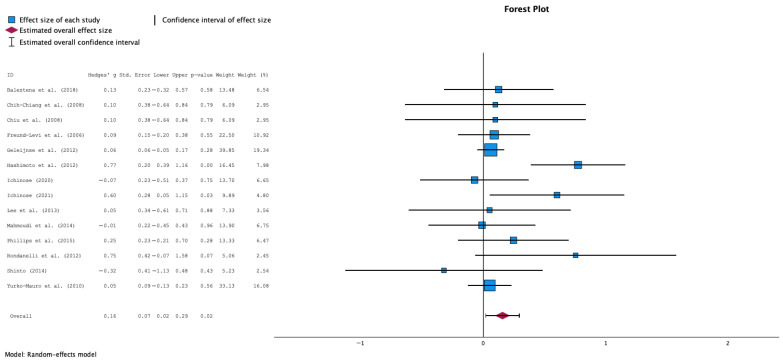
Forest Plot for the overall analysis comparing the effect of n3-PUFA supplementation vs. no supplementation in MMSE scores (random effects model) [31,32,33,34,35,36,37,38,39,40,41,42,43].

This meta-analysis shows a statistically significant overall positive effect of supplementation intervention. The forest plot (Figure 2) reveals the individual study effect sizes and their respective confidence intervals, providing a comprehensive visualization of the evidence base. The analysis included 14 individual studies, with effect sizes ranging from negative values in some studies (Ichinose 2020 [36]: −0.07, Shinto 2014 [42]: −0.32) to strongly positive effects in others (particularly Hashimoto et al. 2012 [35] showing the largest positive effect).

The pooled effect size from the random-effects analysis was 0.16 (95% CI: 0.01 to 0.32), indicating a small but statistically significant positive effect of supplementation on MMSE scores. An increase on MMSE means better cognitive function, while a decrease suggests a deterioration of cognitive function.

The consistency between random-effects and fixed-effects results (data now shown) provides robust evidence for the supplementation’s therapeutic benefit. When both analytical approaches yield similar conclusions, it suggests that the treatment effect is stable and not heavily dependent on the choice of statistical model. This convergence of results strengthens confidence in the meta-analytic findings and supports the clinical relevance of the observed effects.

### 3.3. Heterogeneity Assessment

**Figure 3:**
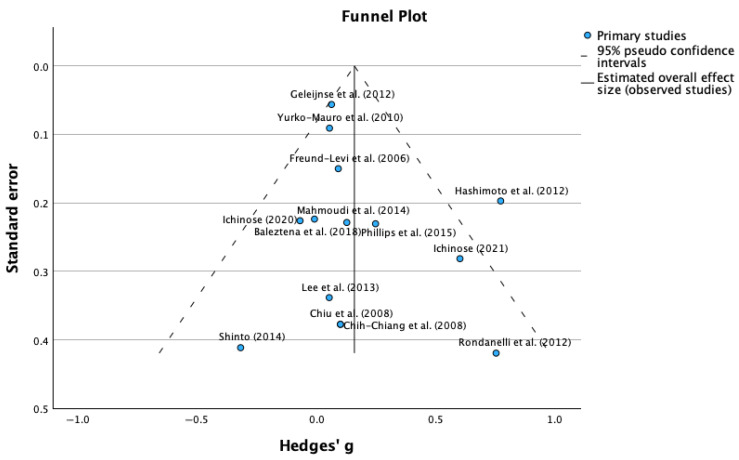
Funnel plot for mean differences in MMSE scores (publication bias assessment) [31,32,33,34,35,36,37,38,39,40,41,42,43].

The heterogeneity analysis revealed low to moderate between-study variability, supporting the suitability of conducting a meta-analysis with these studies (Figure 3). The heterogeneity statistics showed a tau-squared (τ^2^) of 0.022 m, which is relatively low and indicates a minimal heterogeneity between studies, and a I-squared (I^2^) of 42.8%, which represents the percentage of total variation across studies due to heterogeneity rather than chance. Hence, a value of 42.8% indicates moderate heterogeneity according to conventional thresholds; also, an H-squared (H^2^) value of 1.748 was obtained, showing the comparison between the observed variation to what would be expected by chance alone. Values close to 1.0 indicate low heterogeneity.

### 3.4. Egger’s Test for Publication Bias

Complementing the visual funnel plot assessment, Egger’s regression-based test provided quantitative evaluation of publication bias. The statistical analysis revealed an intercept of 0.066 (95% CI: −2.232 to 2.364), a standard error of 1.168, a t-value of 0.465, and a *p*-value of 0.617. The non-significant *p*-value (0.617) from Egger’s test indicates no statistically significant evidence of publication bias. The intercept value close to zero (0.066) and its confidence interval including zero further support the absence of systematic bias in the included studies. The Egger’s test suggests that the literature search strategy successfully identified a representative sample of relevant studies, including those with varying effect sizes and significance levels, and the absence of detected publication bias enhances confidence that the observed positive effects of supplementation reflect true therapeutic benefits rather than selective reporting of favorable outcomes.

### 3.5. Duration–Effect Relationship Analysis

Analysis was conducted to examine the relationship between treatment duration and supplementation effectiveness using Pearson correlation analysis. The investigation assessed whether longer treatment periods were associated with greater therapeutic benefits. In a sample size of 14 RCTs, we obtained a correlation coefficient of 0.038 (*p* = 0.897). The correlation analysis demonstrated no significant relationship between treatment duration (measured in months) and effect size (Hedges’ g), suggesting that the treatment duration does not predict therapeutic outcomes within the range of duration examined across studies.

Studies are distributed across various durations from approximately 6 months to over 36 months, with effect sizes ranging from negative to strongly positive values regardless of treatment length (3 months, 6 months, and 12 months). A scatter plot of categorical duration analysis shows studies are distributed across all three duration categories with comparable effect size variability in each group, further supporting the absence of a duration-dependent treatment effect (Figure S1 and S2—Supplementary Results).

### 3.6. Dose–Response Relationship Analysis

#### Combined DHA + EPA Analysis

This analysis of EPA and DHA intake suggests that reported cognitive benefits may occur across a range of dosages. However, variability in intervention definitions and intake sources limits the ability to draw firm conclusions about dose dependency. These findings suggest that the therapeutic mechanism may involve threshold effects rather than linear dose–response relationships, with benefits achieved once minimum effective levels are reached (Figures S1 and S2—Supplementary Data).

DHA and EPA dosage were examined independently through Pearson correlation analysis to determine whether higher doses were associated with superior therapeutic outcomes (Figure S3—Supplementary Data). The analysis revealed no significant correlation between doses and the therapeutic effect size. The weak negative correlation coefficient (−0.134) with a non-significant *p*-value (0.647) indicates that within the range of DHA dosages examined across included studies, higher doses did not necessarily produce superior therapeutic outcomes compared to lower doses. The same results were shown for EPA. Also, the scatter plot of effect size (Hedges’ g) by DHA and EPA dose (mg) demonstrates the lack of dose–response relationship (Figure S3—Supplementary Data). Studies are distributed across a range of DHA doses from approximately 200 mg to 2000 mg, and of EPA from 0 to 1080 mg, with effect sizes showing no clear pattern related to dosage. Both high and low doses appear capable of producing positive, negative, or neutral effects, confirming the absence of a linear dose–response relationship.

## 4. Discussion

We aimed to determine the effectiveness of oral supplementation with n3-PUFAs as compared to no supplementation in improving cognitive function in patients with MCI. We synthesized the evidence encompassed in 9 SRs, including 14 RCT studies that met our eligibility criteria. This overview of identifies a consistent association between n3-PUFA supplementation and cognitive outcomes across studies, despite the heterogeneity in dosage and duration of the intervention, thus making room for more tailored nutritional recommendations [14,18,44]. By synthesizing evidence from multiple reviews, and by pooling data from RCTs that conducted a standardized and validated assessment of cognitive impairment in a second step, these findings suggest that the observed benefits of supplementation are unlikely to be due to chance alone and represent a genuine therapeutic effect.

PUFAs, and especially EPA and DHA, have long been associated with brain health due to their structural and regulatory roles in neuronal membranes and anti-inflammatory effects [45]. DHA constitutes up to 40% of the polyunsaturated lipids making up brain cells, as well as playing crucial roles in neurotransmission and neuroprotection, paving the way for its use in several clinical trials [46,47].

The beneficial mechanism of action of N3 fatty acids (and especially DHA) is the ability to control the inflammatory process, modifying the fluidity and composition of cell membranes through direct effects on receptor function and the conductance of ion channels involved in immune activation [33]. It is currently accepted that deficient production of anti-inflammatory mediators might be involved in the impairment of the cognition [48]. The deficient production is mainly significant before the onset of dementia during which an increase is observed in pro-inflammatory mediators in the brain. Therefore, it is particularly beneficial to supplement with n3 for the prevention or slowing of the progression of the disease.

Although n3 PUFAs have demonstrated neuroprotective properties through anti-inflammatory, antioxidant, and membrane-stabilizing mechanisms, their impact on clinical outcomes for older adults with cognitive impairment remain inconsistent. Variability may stem from differences in dosage, intervention duration, baseline nutritional status, and genetic factors such as APOE genotype. Additionally, heterogeneity in study populations—ranging from mild cognitive impairment to established dementia—confuses clear interpretation. Cognitive benefits may depend on early intervention, adequate bioavailability, and synergistic dietary factors. All these factors reinforce the need for future trials that must incorporate stratified designs, biomarker monitoring, and longer follow-up to clarify therapeutic windows and optimize personalized strategies for n3 PUFA supplementation in cognitive decline.

Our findings suggest a positive association between *n*-3 PUFA supplementation and cognitive outcomes across studies with varied dosages and durations. While this contrasts with prior literature emphasizing dose and duration as key factors, the lack of standardized intervention definitions warrants cautious interpretation. In fact, many RCTs have found null results regarding *n*-3 PUFA effect, and one meta-analysis has shown that DHA supplementation below 580 mg/day was ineffective [49].

The observed benefits of n3 PUFA supplementation in some studies with lower doses and shorter durations highlight the need for further investigation into threshold effects and underscore the importance of precise intervention characterization. Indeed, there are important differences within the RCTs and meta-analyses regarding the level of cognition among the population (healthy, MCI, and AD), the genetic background (e.g., APOE ε4 status has gained increasing importance) [24], the type of supplementation regimens (EPA vs. DHA ratio), and which is the chosen cognitive outcome measure (e.g., MMSE, ADAS-Cog, memory vs. executive tests).

There are some biological mechanisms that might explain why the effect could be independent of time or dose. Indeed, brain DHA uptake relies on transporters (e.g., MFSD2A) that work in a saturation-dependent manner. Once they are saturated, DHA can no longer confer additional benefits [50]. Interestingly, in cognitively healthy individuals, 3.36 g EPA and DHA daily slowed cognitive aging by 2.5 years, although there were observed differences amongst the RCTs. As expected, the potential mechanisms explaining differences in the outcomes included n3PUFA dose, trial duration, apolipoproteinE genotype, and the downstream product of DHA neuroprotectin D1, which is suggested to be involved in beneficial effects and is determined by integrated biology rather than milligram dose [51,52]. This supports the notion that baseline status and genetics (e.g., APOE ε4) may influence how quickly individuals reach these effective thresholds. Additionally, once n3 levels reach optimal cellular concentration, the brain may retain these lipids irrespective of continued high-dose supplementation, enabling sustained benefit. Interestingly, a recent article has highlighted the substantial influence of the genetic variants of APOE4 homozygosity on plasma metabolites and dementia risk [53]. These findings highlight the potential for preventive strategies targeting specific metabolic pathways in this high-risk group (APOE4 homozygotes), with a long-term adherence to the Mediterranean diet which is known for fish and rich n3-PUFA food items.

Indeed, some epidemiological studies report a reduction in the risk of cognitive impairment with the intake of fish rich in n3 fatty acids, although results do not show an overall improvement and are limited only to certain aspects of cognitive function in patients with cognitive impairment not associated with dementia [29,54,55]. The absence of detailed data on usual diet and baseline *n*-3 PUFA dietary intake in the present meta-analysis restricts interpretation, as these factors might either mask supplementation effects or synergistically enhance them. Moreover, variability in the diet, absorption efficiency, and nutrient interactions may further complicate outcome evaluation, hence why future studies ought to integrate dietary assessments and biomarkers of *n*-3 PUFA status to clarify the independent and combined contributions of diet and supplementation.

The consistent positive association, independent of dosage or exposure duration, indicates that *n*-3 PUFA confers neurocognitive resilience through threshold-dependent mechanisms. Once critical plasma or neural membrane concentrations are achieved, they facilitate synaptic stability, attenuate neuroinflammation, and optimize signaling, emphasizing biomarker thresholds over linear dose–response or time-dependent effects. This paradigm shift enhances efficiency, minimizes unnecessary consumption, and aligns nutritional advice with individual physiology—marking a significant milestone in our understanding of *n*-3 PUFA role in cognitive health and opening pathways to tailored recommendations that are evidence-based, sustainable, and personalized.

By providing an overview of the most recent SRs (published since 2014) on the topic of n3 and cognitive impairment, this study has several strengths. Usually, these SRs cannot be meta-analyzed due to the way results are reported and the use of a variety of methods to assess cognitive impairment. However, we managed to select trials that used the validated and widely recommended method to assess cognitive impairment, allowing us to conduct a series of meta-analyses to determine the overall effect of n3 PUFA supplementation strategy on cognitive impairment as well as the potential effect of dose and duration of the supplementation. We also followed the recommended PRISMA reporting guidelines to identify, select, critically appraise, and synthetize SRs. Importantly, our study does not allow us to suggest a specific formulation of PUFAs to prevent cognitive decline and future research with dose–response trials will be needed to draw more firm conclusions about the ideal *n*-3 formulation.

Our study contains some limitations. First, as stated above, we only studied the effect of supplementation, not other sources of *n*-3 PUFAs. In fact, the lack of dose–response effects could be explained by other confounding factors such as different baseline levels of n3-PUFAs, other dietary sources of n3-PUFAs consumed during the study, and other co-interventions. However, these RCTs did not provide any additional information regarding diet and nutritional intake. Second the quality of the SRs included is variable, with two out of seven being assessed as critically low. Third, as we excluded some trials that did not assess cognitive impairment with the MMSE (a condition to perform meta-analyses), it is possible that those trials might have observed different findings [45]. Nonetheless it is important to highlight that there was not a similar tool to evaluate cognitive impairment, or subjective memory complaints, across all the RCTs/systematic reviews, hence the need to rely on the MMSE as the most used tool to evaluate the outcome. Fourth, the use of different types of oils (e.g., coconut oil, olive oil, soybean oil), some of which contain significant levels of *n*-3 long chain PUFA precursor 18:3 *n*-3 (linolenic acid), may have an influence on the assessed outcome.

## 5. Conclusions

The observed positive (albeit small) effect of n3 supplementation on reduction in cognitive decline, independent of time or dose, suggests that clinicians can consider using this intervention paired with other recommended interventions to prevent cognitive impairment. This may include regular physical activity, healthy diet (high in vegetables, fruits, whole grains, legumes, fish, and healthy fats; low in red meats and ultra processed foods), sleep optimization, avoidance of neurotoxic substances, cognitive training, and mental stimulation.

## Figures and Tables

**Table 1:** Inclusion and exclusion criteria.

Criteria	Inclusion Criteria	Exclusion Criteria
Population	Adults or older adults, healthy or with MCI, mild cognitive decline, Alzheimer’s Disease	Age < 18, pregnant or breastfeeding women, or individuals with diagnosed depression
Intervention	Supplementation of exclusively *n*-3 fatty acids	Supplementation with other nutrients (in addition to *n*-3 fatty acids)
Comparators	Control or treatment	
Outcome	Objective and measurable effect on cognitive functional with MMSE tool	Studies with no detailed outcome indicators
Timing	Any duration of the intervention	
Setting	Hospitalized, living in nursing homes or at home	
Study design	SR of RCT	Quasi-RCTs, cluster RCTs, animal studies, case studies, qualitative studies, conference abstracts, comments, letters and duplicated articles, and lack of research information
Other	English, Spanish, Portuguese languages	

Abbreviations: MCI = mild cognitive impairment, SR = systematic review, RCT = randomized controlled trial, MMSE = mini mental state examination.

**Table 2:** Characteristics of includes SRs.

Author Year	Objective	Number of RCT	Disease	N	Population	Intervention	Comparison	Time Intervention
Cynthia Batista Santos 2024 [24]	To discuss the effects of DHA and EPA supplementation on cognitive decline variables and relate them to APOEε4 genotype in middle-aged and older adults	2	Healthy and Probable diagnosis of mild AD	665 (I: 355; C: 310)	74.6 years	1720 mg–2 g DHA; 0–600 mg EPA	Corn/soybean oil; 990 mg of olive oil + 10 mg of fish oil (1.2 mg DHA + 1.8 mg EPA)	18 months
Xiaoling Zhang 2019 [25]	To assess the effects of *n*-3 LC-PUFAs intake on MCI patients to explore whether *n*-3 LC-PUFAs have positive effective	7	MCI	434 (I: 213; C: 221)	74.8 years	180–1300 mg DHA; 40–720 mg EPA	-	3–24 months
Anu Alex 2019 [17]	To determine the changes in cognitive function after intervention with LCn-3PUFA supplementation in non-demented adults, including those with MCI	25	Healthy and MCI	6981 (I: 3530; C: 3451)	57.54 years	180–1550 mg DHA; 0–2187 mg EPA	-	4 weeks–5 years
Lei Yang 2023 [26]	To investigate the effectiveness of DHA and EPA supplements in the elderly with MCI.	12	MCI	1124 (I: 558; C: 566)	≥60 years	180–2000 mg DHA; 0–1080 EPA	-	2–24 months
Amelia Martí 2019 [27]	To determine whether there is or not a positive effect of *n*-3 LCPUFAs supplementation on cognitive decline.	14	Healthy adults, cognitive impairment/complaints, MCI, cognitive impairment no dementia or AD	1638	>45 years	252–2000 mg DHA; 0–1600 EPA; Krill oil (193 mg EPA + 92 mg DHA) or sardine oil (491 mg EPA + 251 mg DHA); 120 mg ARA	-	1–12 months
Xiao-Wei Zhang [28]	To assess the association between omega-3 fatty acids and risk of cognitive decline in the elderly	6	Healthy	2013 (I: 1003; C: 1010)		240–900 mg DHA; 0–1080 mg EPA	-	3–40 months
Marion Burckhardt 2016 [29]	To assess the efficacy and safety of *n*-3 PUFA supplementation for the treatment of people with dementia	3	AD, PD, vascular dementia, dementia with Lewy bodies, and frontotemporal dementia	632 (I: 354; C: 278)	74.8 years	675 mg–1.7 g DHA; 0–975 mg EPA	Isocaloric placebo oil (1 g corn oil, including 0.6 g linoleic acid) and 4 mg vitamin E; soy oil; soybean with 5% fish oil	6–18 months
Xin He 2023 [30]	To evaluate the effect of *n*-3 PUFA on cognitive function in the elderly and the role of baseline omega-3 index	15	Healthy, cognitive impairment	3734 (I: 1906; C: 1828)	>60 years	160–2000 mg DHA; 0–975 mg EPA	Linoleic acid; olive oil esters; corn oil; soy oil; margarine; soybean oil; coconut oil; olive oil, 30 mg EPA + 106 mg DHA; paraffin oil	4–40 months
Seung Wan Suh 2024 [14]	To examine the effect of *n*-3 PUFA on the cognitive function of middle-aged or older adults without dementia	24	Healthy	9660 (I: 4834, C: 4826)	>40 years	230 to 4000 mg PUFA		3–36 months

Abbreviations: RCTs = randomized controlled trials; N = number; DHA = docosahexaenoic acid; EPA = eicosapentaenoic acid; AD = Alzheimer’s disease; I = intervention group; C = control group; y = year; m = month; *n*-3 LC-PUFAs = N-3 long chain polyunsaturated fatty acids; MCI = mild cognitive impairment; ARA = arachidonic acid; PD = Parkinson’s disease.

**Table 3:** Outcomes, results, and studies quality assessment of the included systematic reviews.

Author Year	Outcomes	Results	Amstar 2 Quality
Cynthia Batista Santos 2024 [24]	MMSE without pooled results	DHA and EPA supplementation showed no pragmatic effects on cognitive variables when considering the presence of the APOEε4 allele.	Moderate
Xiaoling Zhang 2019 [25]	Intervention: 24.85 MMSE; Control: 23.89 MMSE	N-3 LC-PUFA may have beneficial effects in the elderly with MCI.	Low
Anu Alex 2019 [17]	MMSE without pooled results	N-3 LC-PUFA supplementation could provide a mild benefit in improving memory function in nondemented older adults	High
Lei Yang 2023 [26]	Intervention: 24.19 MMSE; Control: 23.38 MMSE	DHA and/or EPA supplements have benefits on global cognition, and it may also reduce the level of blood amyloid-β related biomarkers and inflammatory factors.	Moderate
Amelia Martí 2019 [27]	MMSE without pooled results	Omega-3 supplementation might have a positive effect on cognitive function. Thus, *n*-3 LCPUFAs could be used as a preventive or therapeutic tool for cognitive decline in aged or elder adults.	Critically low
Xiao-Wei Zhang [28]	Intervention: 26.18 MMSE; Control: 21.4 MMSE	Omega-3 fatty acids may help to prevent cognitive decline in the elderly.	Critically low
Marion Burckhardt 2016 [29]	Intervention: 20.85 MMSE; Control: 25.6 MMSE	No evidence for either benefit or harm from N-3 LC-PUFA supplements in people with mild to moderate Alzheimer’s disease.	High
Xin He 2023 [30]	MMSE without pooled results	N-3 LC-PUFA supplementation exerted no improvement on global cognitive function. However, a higher baseline omega-3 index and higher omega-3 index increment were associated with an improvement in cognitive function in the elderly.	Moderate
Seung Wan Suh 2024 [14]	MMSE without pooled results	Supplementation of N-3 LC-PUFA may offer potential advantages for executive function in the middle-aged and elderly population, particularly in individuals whose dietary DHA + EPA level is not substantially diminished.	High

Abbreviations: MMSE = mini mental state examination; DHA = docosahexaenoic acid; EPA = eicosapentaenoic acid; *n*-3 LC-PUFAs = N-3 long chain polyunsaturated fatty acids; MCI = mild cognitive impairment. Description of the included systematic reviews.

**Table 4:** Characteristics of RCTs included.

Study (RCT)	N (T/C)	Disease	Age	Intervention	Comparison	Time (Months)	Results
Baleztena et al. (2018) [31]	78 (34/44)	Healthy or with MCI	≥86.90	40 mg EPA + 250 mg DHA	No treatment or basic treatment	12	No effect
Chih-Chiang et al. (2008) [32]	29 (17/12)	Mild or moderate Alzheimer’s or MCI	74	720 mg EPA + 1080 mg DHA	Olive oil esters	6	Positive effects of intervention
Chiu et al. (2008) [32]	29 (17/12)	MCI	73	1080 mg EPA + 720 mg DHA	Olive oil esters	6	Positive effects of intervention
Freund-Levi et al. (2006) [33]	178 (91/87)	MCI	72.6	600 mg EPA + 1600 mg DHA	Linoleic acid	6	Positive effects in a small group of patients with very mild AD
Geleijnse et al. (2012) [34]	1265 (627/638)	Healthy	69	160 mg EPA + 240 mg DHA	Margarine	40	No effect
Hashimoto et al. (2012) [35]	111 (57/54)	CI	72	470 mg EPA + 1270 mg DHA	-	24	Positive effects of intervention
Ichinose (2020) [36]	79 (43/36)	Healthy	73	137 mg EPA + 297 mg DHA	Soybean oil	12	Positive effects of intervention
Ichinose (2021) [37]	53 (26/27)	Healthy	69.1	137 mg EPA + 297 mg DHA	Normal milk	12	Prevented age-related cognitive decline
Lee et al. (2013) [38]	35 (17/18)	MCI	≥60	450 mg EPA + 1300 mg DHA	Corn oil	12	Positive effects of intervention
Mahmoudi et al. (2014) [39]	80 (40/40)	Healthy and MCI	74.1	120 mg EPA + 180 mg DHA	Coconut oil	6	No effect
Phillips et al. (2015) [40]	76 (37/39)	MCI	71.1	600 mg EPA + 625 mg DHA	Olive oil	4	No effect
Rondanelli et al. (2012) [41]	25 (11/14)	MCI	86	286 mg EPA + 720 mg DHA	-	3	Positive effects of intervention
Shinto (2014) [42]	24 (12/12)	MCI	75.6	975 mg EPA + 675 mg DHA	Soybean oil	12	Positive effects of intervention
Yurko-Mauro et al. (2010) [43]	485 (242/243)	Healthy	70	900 mg DHA	Corn oil, soy oil	6	Positive effects of intervention

Abbreviation: RCTs = randomized controlled trials; N = number T = number treatment; C = number control; MCI = mild cognitive impairment; DHA = docosahexaenoic acid; EPA = eicosapentaenoic acid; AD = Alzheimer disease; CI: cognitive impairment. (References: [31,32,33,34,35,36,37,38,39,40,41,42,43]).

**Table 5:** Mean (SD) scores of MMSE in the treatment and control groups of the included RCTs.

Study (RCT)	Treatment		Control	
	NT	MeanT ± SDT	NC	MeanC ± SDC
Baleztena et al. (2018) [31]	34	23.67 ± 4.95	44	23.00 ± 5.54
Chih-Chiang et al. (2008) [32]	17	25.47 ± 3.81	12	25.09 ± 3.67
Freund-Levi et al. (2006) [33]	91	22.80 ± 4.38	87	22.40 ± 4.52
Geleijnse et al. (2012) [34]	627	27.70 ± 1.75	638	27.60 ± 1.52
Hashimoto et al. (2012) [35]	57	28.80 ± 2.00	54	27.00 ± 2.60
Ichinose (2020) [36]	43	27.40 ± 4.50	36	27.70 ± 3.70
Ichinose (2021) [37]	26	29.5 ± 0.8	27	28.8 ± 1.4
Lee et al. (2013) [38]	17	26.60 ± 1.87	18	26.50 ± 1.93
Mahmoudi et al. (2014) [39]	40	17.81 ± 1.71	40	17.83 ± 1.97
Phillips et al. (2015) [40]	37	24.40 ± 4.10	39	23.30 ± 4.70
Rondanelli et al. (2012) [41]	11	27.18 ± 1.80	14	24.56 ± 4.18
Shinto (2014) [42]	12	18.90 ± 4.40	12	20.40 ± 4.60
Yurko-Mauro et al. (2010) [43]	242	28.00 ± 1.90	243	27.90 ± 1.90
Chiu et al. (2008) [32]	17	25.47 ± 3.81	12	25.09 ± 3.67

Abbreviation: RCTs = randomized controlled trials; NT = number treatment; MeanT = mean treatment; SDT = standard deviation treatment; NC = number control; MeanC = mean control; SDC = standard deviation control; (References: [31,32,33,34,35,36,37,38,39,40,41,42,43]).

## Data Availability

No new data were created or analyzed in this study. Data sharing is not applicable to this article.

## Supplementary Materials

The following supporting information can be downloaded at: https://www.mdpi.com/article/10.3390/nu17183002/s1. Figure S1. DHA and EPA Dose-response relationship to the duration in moths, Figure S2. DHA and EPA Dose-response relationship to the duration in categories, Figure S3. DHA and EPA Dose-effect analysis, Figure S4. DHA and EPA combined Dose-effect analysis.

## Abbreviations

The following abbreviations are used in this manuscript:

MCI	Mild Cognitive Impairment
DHA	Docosahexaenoic Acid
EPA	Eicosapentaenoic acid
RCT	Randomized Controlled Trial
MMSE	Mini Mental State Examination
PUFAs	Polyunsaturated Fatty Acids
PRISMA	Preferred Reporting Items for Systematic Reviews and Meta-Analyses
SR	Systematic Review
ADAS-cog	Assessment Scale-Cognitive Subscale
