# Supplementary material for: Omega-3 Polyunsaturated Fatty Acids and Cognitive Decline in Adults with Non-Dementia or Mild Cognitive Impairment: An Overview of Systematic Reviews
Source: Nutrients. 2025 Sep 19;17(18):3002. doi: 10.3390/nu17183002 (PMC12472900; doi:10.3390/nu17183002)
Supplement: Supplementary file 1 [file nutrients-17-03002-s001.zip › nutrients-3790303-supplementary.pdf]

Dose-Response Relationship Analysis

<u>*Dose-duration*</u>

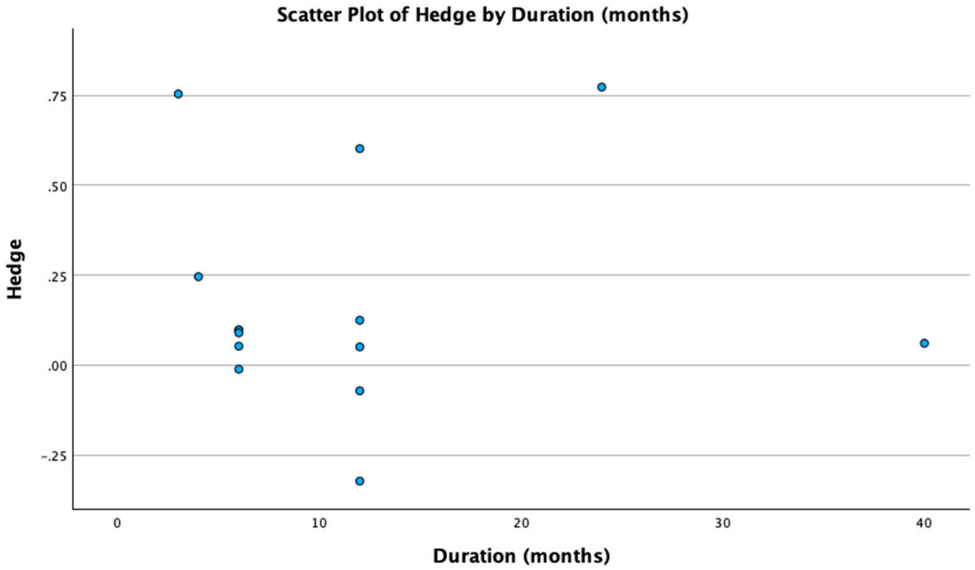

Figure S1 – DHA and EPA Dose-response relationship to the duration in months

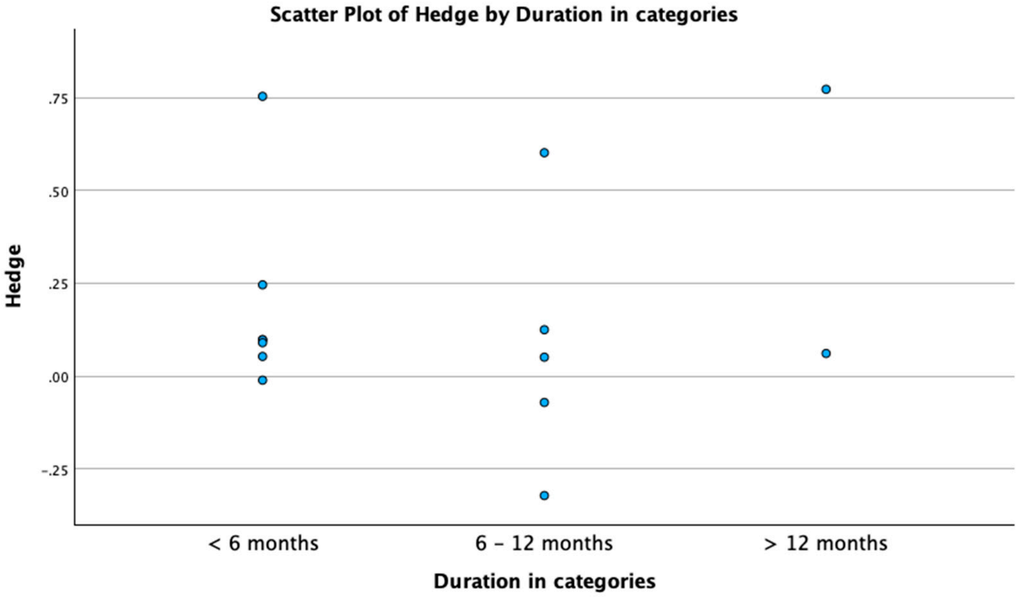

Figure S2 - DHA and EPA Dose-response relationship to the duration in categories

Dose-effect

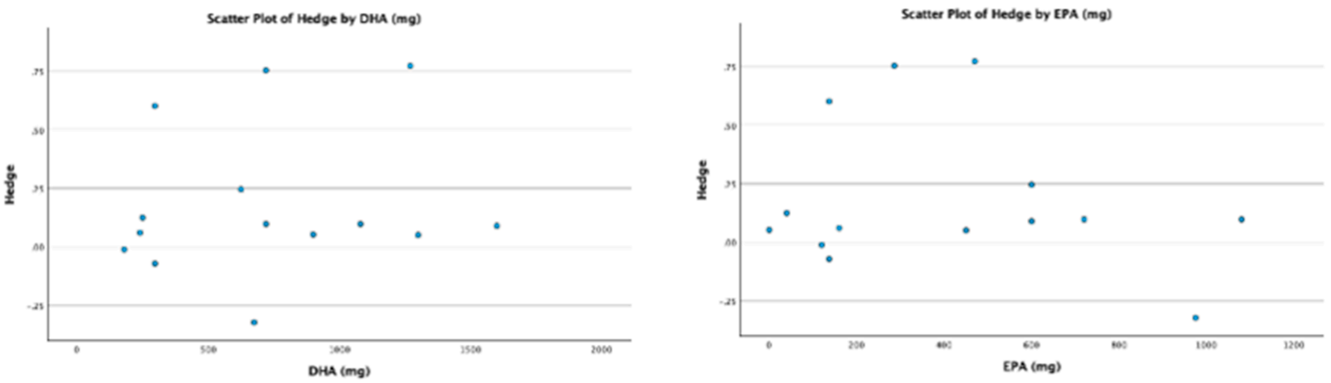

Figure S3 - DHA and EPA Dose-effect analysis

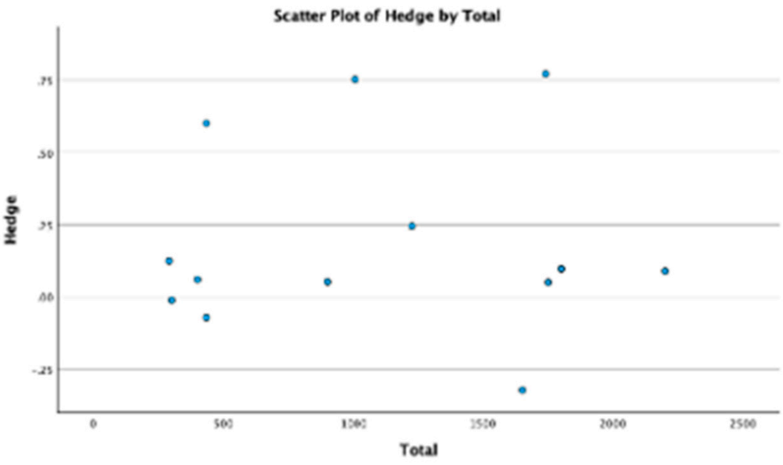

Figure S4 – DHA and EPA combined Dose-effect analysis
